# 伴TP53基因突变急性B淋巴细胞白血病的临床特征及预后分析

**DOI:** 10.3760/cma.j.issn.0253-2727.2021.05.008

**Published:** 2021-05

**Authors:** 园园 杜, 康康 吕, 蜜蜜 徐, 卫芹 姚, 慧珠 康, 悦 韩, 晓文 唐, 骁 马, 小津 吴, 雪峰 何, 德沛 吴, 跃均 刘

**Affiliations:** 苏州大学附属第一医院、江苏省血液研究所、国家血液系统疾病临床医学研究中心、苏州大学造血干细胞移植研究所、血液学协同创新中心 215006 National Clinical Research Center for Hematologic Diseases, Jiangsu Institute of Hematology, The First Affiliated Hospital of Soochow University, Institute of Blood and Marrow Transplantation, Collaborative Innovation Center of Hematology, Soochow University, Suzhou 215006, China

**Keywords:** 白血病，B淋巴细胞，急性, 基因，TP53, 异基因造血干细胞移植, 嵌合抗原受体, Leukemia, B lymphoblastic, acute, Gene, TP53, Allogenetic hematopoietic stem cell transplantation, Chimeric antigen receptors

## Abstract

**目的:**

研究TP53基因突变阳性急性B淋巴细胞白血病（B-ALL）患者的临床特征及预后。

**方法:**

回顾性分析2016年1月至2019年12月在苏州大学附属第一医院治疗的479例初诊B-ALL患者的临床资料。

**结果:**

479例B-ALL患者中，34例（7.1％）TP53基因突变阳性，共检测到36个TP53突变，其中移码基因突变10个（27.8％），错义突变23个（63.9％），无义突变3个（8.3％）。共有34个（94.4％）突变位于DNA结合结构域（第5～8号外显子）。伴TP53基因突变组患者平均突变基因数目（2.3个）与无TP53基因突变组（1.1个）差异有统计学意义（*P*<0.001）。Ph阳性和Ph-like阳性患者在TP53基因突变阴性组中的比例显著高于TP53突变阳性组，差异有统计学意义（*P*<0.001）。TP53基因突变阴性组3年总生存（OS）率、无事件生存（EFS）率显著高于TP53基因突变阳性组（*χ*^2^＝4.694，*P*＝0.030；*χ*^2^＝5.080，*P*＝0.024）。多因素分析中，1个疗程诱导化疗未完全缓解（CR）是影响患者OS的独立预后不良因素。34例伴TP53基因突变患者中16例在第1次CR（CR_1_）状态行异基因造血干细胞移植（allo-HSCT），2例移植后复发输注供者来源的抗CD19嵌合抗原受体T（CAR-T）细胞后获CR_2_。11例巩固化疗过程中复发的TP53基因突变患者中6例行抗CD19 CAR-T细胞治疗，4例获得缓解且微小残留病（MRD）转阴，缓解后桥接allo-HSCT，其中2例持续CR。

**结论:**

伴TP53基因突变B-ALL患者中错义突变最常见，突变位点主要分布于DNA结合结构域。伴TP53基因突变的B-ALL患者复发后CAR-T细胞治疗清除MRD后应尽早行allo-HSCT。伴TP53基因突变的B-ALL患者在allo-HSCT后仍有较高的复发率，输注供者来源的CAR-T细胞能获得较好的持续缓解。

## Abstract (en)

**Objective:**

To investigate the survival and prognosis of B-lineage acute lymphoblastic leukemia（B-ALL）patients with TP53 mutation.

**Methods:**

The clinical data of 479 newly diagnosed BALL patients treated in the First Affiliated Hospital of Soochow University from January 2016 to December 2019 were retrospectively analyzed.

**Results:**

Among 479 B-ALL patients, 34 cases（7.1％） were positive for TP53 gene mutation, and a total of 36 TP53 mutations were detected, including 10 frameshift gene mutations（27.8％）, 23 missense mutations（63.9％）and 3 nonsense mutations（8.3％）. A total of 34（94.4％）mutations were located in the DNA binding domain（exons 5–8）.The average number of mutated genes in patients with TP53 gene mutation（2.3）and the group without TP53 gene mutation （1.1）were statistically different（*P*<0.001）. The proportion of Ph positive and Ph-like positive patients in the TP53 gene mutation negative group was significantly higher than that of the TP53 mutation positive group, and the difference was statistically significant（*P*<0.001）. The 3-year OS and EFS rates of the TP53 gene mutation negative group were significantly higher than those of the TP53 gene mutation positive group. The differences in OS and EFS rates between the two groups were statistically significant（*χ*^2^=4.694, *P*=0.030; *χ*^2^=5.080, *P*=0.024）. In the multivariate analysis, failure to achieve remission（CR）after one course of induction chemotherapy was an independent adverse prognostic factor affecting OS.Of the 34 patients with TP53 mutation, 16 underwent allogeneic hematopoietic stem cell transplantation（allo-HSCT） in the CR_1_ state, and 2 patients with recurrence after transplantation obtained CR_2_ after infusion of donorderived anti-CD19 chimeric antigen receptor T（CAR-T）cells. Among the 11 patients with TP53 gene mutation who relapsed during consolidation chemotherapy, 6 received anti-CD19 CAR T cell therapy, 4 patients achieved remission and minimal residual disease（MRD）turned negative, followed by bridging allo-HSCT, and 2 of them sustained CR.

**Conclusion:**

Missense mutations are the most common in B-ALL patients with TP53 gene mutation, and the majority of mutations were located in the DNA binding domain. B-ALL patients with TP53 gene mutation should undergo allo-HSCT as soon as possible after CAR-T cell therapy has cleared the MRD after recurrence. B-ALL patients with TP53 gene mutation still have a higher recurrence rate after allo-HSCT, and the infusion of donor-derived CAR-T cells can achieve better sustained remission.

急性B淋巴细胞白血病（B-ALL）是一种侵袭性的B细胞来源的恶性克隆性疾病，在生物学和临床上均具有很大的异质性。近年来，二代测序（NGS）作为新的分子生物学技术，在探索血液肿瘤的分子发病机制及指导临床诊疗方面发挥了重要作用。TP53作为人类恶性肿瘤中常见的突变基因，可见于各种实体肿瘤和血液恶性肿瘤中，通常与疾病进展和预后不良有关[1]。研究表明，伴TP53基因突变是B-ALL患者疾病早期复发和总生存（OS）时间短的独立危险因素[2]。在本研究中，我们回顾性分析479例B-ALL患者的临床资料，进一步探讨伴TP53基因突变B-ALL患者的临床特征及预后，并分析影响患者预后的危险因素。

## 病例与方法

1. 病例资料：回顾性分析34例2016年1月至2019年12月在苏州大学附属第一医院行NGS检出的TP53突变阳性的B-ALL患者的临床资料。所有患者的诊断均符合文献[3]标准，并根据骨髓细胞形态学、免疫表型分析、细胞遗传学、分子生物学（MICM）进行诊断分型。

2. NGS分析：提取患者骨髓单个核细胞，抽提基因组DNA，构建包括NRAS、FLT3、ASXL1、DNMT3A、TP53等51个血液病相关常见热点基因的Ion AmpliSeq文库，使用ABI Ion Torrent S5测序仪进行检测。

3. 诱导化疗方案：34例TP53突变阳性患者中，30例接受IVP±CTX±L-Asp（去甲氧柔红霉素+长春新碱+泼尼松±环磷酰胺±左旋门冬酰胺酶）诱导化疗方案，4例接受Hyper-CVAD（环磷酰胺+长春新碱+盐酸多柔比星+地塞米松）诱导化疗方案。对Ph^+^B-ALL患者诱导化疗时加用一代或二代酪氨酸激酶抑制剂。

4. 造血干细胞移植（HSCT）：34例TP53突变阳性患者中，共有26例接受HSCT。均采用改良BuCy（白消安+环磷酰胺）±抗胸腺细胞球蛋白（ATG）的预处理方案。接受同胞HLA全相合移植的患者使用甲氨蝶呤（MTX）联合环孢素A（CsA）的方案预防移植物抗宿主病（GVHD），接受无关HLA全相合和单倍型移植的患者使用ATG、MTX、CsA及霉酚酸酯（MMF）的方案预防GVHD。中性粒细胞绝对计数连续3 d>0.5×10^9^/L为粒系造血重建，PLT连续3 d>20×10^9^/L且脱离血小板输注为巨核系造血重建。

5. 疗效判定及随访：参考文献[3]标准判断疗效。随访采用门诊、住院复查或电话方式。随访截止时间为2020年12月1日。OS时间定义为确诊之日至患者因任何原因死亡或末次随访日，无事件生存（EFS）时间定义为确诊之日至血液学复发或死亡日，无上述事件发生者计算至末次随访日。

6. 统计学处理：应用SPSS 23.0软件进行统计学分析。计量资料以中位数（范围）表示，采用Mann-Whitney *U*检验进行比较。计数资料以百分比（％）表示，采用卡方检验进行比较。采用Kaplan-Meier法分析OS及EFS，并应用Log-rank检验进行组间比较。采用Cox风险模型进行影响OS、EFS的单因素分析，*P*<0.1的因素进入Cox回归模型进行多因素分析，*P*<0.05为差异有统计学意义。应用GraphPad Prism8软件绘制统计图。

## 结果

1. TP53基因突变情况：479例B-ALL患者中共检出34例（7.1％）伴有TP53基因突变，TP53中位突变频率（VAF）为26.65％（2.0％～95.4％）。共检测到36个TP53突变，其中移码基因突变10个（27.8％），错义突变23个（63.9％），无义突变3个（8.3％）。其中34个（94.4％）突变位于DNA结合结构域（第5～8号外显子）。出现频率较高的突变位点分别有密码子282、283、248、273、272。伴有TP53基因突变组患者的平均基因突变数目为2.3个，无TP53基因突变组患者的平均基因突变数目为1.1个，差异有统计学意义（*P*<0.001）（图1）。

**图1:**
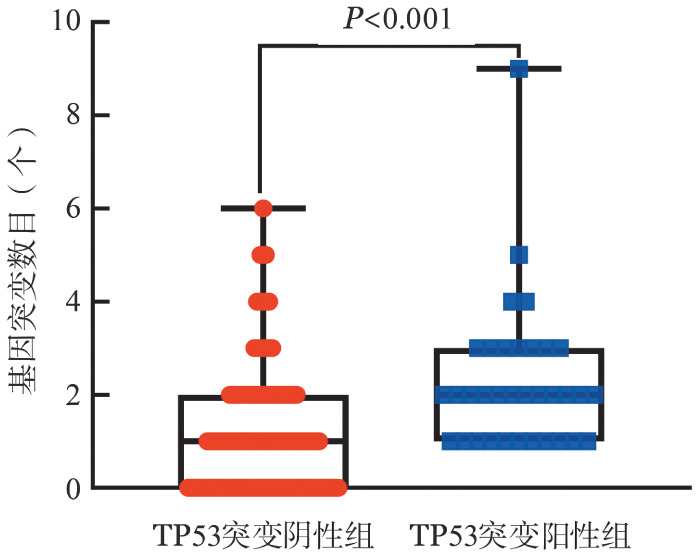
TP53基因突变阳性组（34例）和阴性组（445例）急性B淋巴细胞白血病患者的基因突变数目比较

2. 伴TP53基因突变患者的临床特征：479例B-ALL患者中，TP53突变阴性组445例，其中Ph阳性和Ph-like阳性185例（41.6％）；TP53突变阳性组34例，其中Ph阳性和Ph-like阳性3例（8.8％）。Ph阳性和Ph-like阳性患者在TP53突变阴性组中的比例显著高于TP53突变阳性组，差异有统计学意义（*P*<0.001）。TP53突变阳性组染色体复杂异常及t（4；11）改变比例明显高于TP53突变阴性组，差异有统计学意义（*P*<0.001；*P*＝0.011）。TP53基因突变组阳性与阴性组患者各临床特征之间的比较详见表1。

**表1:** TP53基因突变阳性组与阴性组急性B淋巴细胞白血病患者临床特征比较

临床特征	TP53突变阳性组（34例）	TP53突变阴性组（445例）	统计量	*P*值
性别［例（％）］			0.910	0.340
男	16（47.1）	247（55.5）		
女	18（52.9）	198（44.5）		
年龄［岁，*M*（范围）］	25（9～70）	32（7～74）	4.729	0.030
WBC［×10^9^/L，*M*（范围）］	10.2（2.0～277.0）	16.0（0.3～645.0）	0.818	0.366
HGB［g/L，*M*（范围）］	103.5（38～164）	89（26～166）	2.781	0.095
PLT［×10^9^/L，*M*（范围）］	70（5～376）	48（1～582）	0.400	0.527
骨髓原始幼稚细胞比例［％，*M*（范围）］	84.0（7.0～96.8）	82.0（11.0～99.0）	0.203	0.652
染色体核型分析［例（％）］				
高超二倍体	1（2.9）	6（1.3）	0.557	0.456
t（4；11）	4（11.8）	14（3.1）	6.488	0.011
Ph和Ph-like	3（8.8）	185（41.6）	12.868	<0.001
亚二倍体	0（0）	4（0.9）		–
复杂异常	10（29.4）	41（9.2）	13.545	<0.001
其他	16（47.1）	195（43.8）		–
诱导化疗方案［例（％）］			1.356	0.244
IVP±CTX±L-Asp	30（88.2）	416（93.5）		
Hyper-CVAD	4（11.8）	29（6.5）		
1个疗程诱导化疗疗效［例（％）］			1.742	0.419
CR	31（91.2）	404（90.8）		
PR	2（5.9）	12（2.7）		
NR	1（2.9）	29（6.5）		
移植［例（％）］			0.396	0.529
是	26（76.5）	360（80.9）		
否	8（23.5）	85（19.1）		
复发［例（％）］			5.490	0.019
是	17（50）	136（30.6）		
否	17（50）	309（69.4）		
死亡［例（％）］			3.263	0.071
是	13（38.2）	108（24.3）		
否	21（61.8）	337（75.7）		

注：IVP±CTX±L-Asp：去甲氧柔红霉素+长春新碱+泼尼松±环磷酰胺±左旋门冬酰胺酶；Hyper-CVAD：环磷酰胺+长春新碱+盐酸多柔比星+地塞米松；CR：完全缓解；PR：部分缓解；NR：未缓解

3. 疗效及预后分析：479例B-ALL患者中，TP53突变阴性组445例，1个疗程诱导化疗完全缓解（CR）404例（90.8％）；TP53突变阳性组34例，1个疗程诱导化疗CR 31例（91.2％）。两组患者诱导化疗CR率差异无统计学意义（*P*＝0.419）。

截至2020年12月1日，中位随访时间27（2～59）个月。其中，34例TP53突变阳性组患者复发17例（50％）、死亡13例（38.2％），445例TP53突变阴性组患者复发136例（30.6％）、死亡108例（24.3％），TP53突变阳性组患者复发比例显著高于TP53突变阴性组，差异有统计学意义（*P*＝0.019）。TP53突变阴性组患者3年OS、EFS率分别为（74.0±2.2）％、（57.8±2.5）％，TP53突变阳性组3年OS、EFS率分别为（54.6±12.2）％、（42.0±8.8）％。两组OS、EFS率差异有统计学意义（*χ*^2^＝4.694，*P*＝0.030；*χ*^2^＝5.080，*P*＝0.024）（图2）。

**图2:**
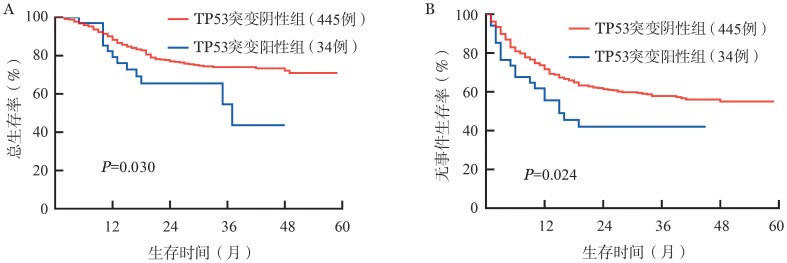
TP53基因突变阳性组与阴性组急性B淋巴细胞白血病患者总生存（A）及无事件生存（B）比较

31例TP53基因突变阳性诱导治疗CR患者中，19例在CR_1_状态下行HSCT（3例auto-HSCT，16例allo-HSCT），4例移植后复发（3例为allo-HSCT后复发），其中2例行供者来源CAR-T细胞治疗再次缓解并长期生存，另外2例再诱导化疗未达缓解死亡。化疗巩固的12例患者中11例复发，其中6例复发后行CAR-T细胞治疗，4例在CAR-T细胞治疗再次获得缓解后桥接allo-HSCT，2例CAR-T治疗后未达缓解死亡；2例在复发后再诱导未缓解状态下行挽救性allo-HSCT；1例入组BITE临床试验；2例病程中仅行化疗。

4. 预后因素分析：本组34例TP53基因突变阳性患者单因素分析结果显示，1个疗程诱导化疗未获CR、未进行移植是影响患者OS的危险因素（表2）。1个疗程诱导化疗未获CR、未进行移植、染色体复杂异常是影响患者EFS的危险因素（表2）。将*P*<0.1的因素纳入Cox回归多因素分析模型，结果表明1个疗程诱导化疗未获CR是影响患者OS的独立预后不良因素（表3）。

**表2:** 伴TP53基因突变急性B淋巴细胞白血病患者预后影响因素的单因素分析

因素	总生存			无事件生存		
	*HR*	95％ *CI*	*P*值	*HR*	95％ *CI*	*P*值
年龄（≤45岁，>45岁）	0.609	0.329～1.127	0.115	0.979	0.564～1.701	0.941
性别（男，女）	1.198	0.388～3.699	0.754	1.041	0.418～2.593	0.932
WBC（>30×10^9^/L，≤30×10^9^/L）	1.503	0.319～7.087	0.607	1.036	0.337～3.186	0.951
染色体核型（复杂异常，其他）	2.247	0.751～6.726	0.148	2.552	1.032～6.312	0.043
伴随突变基因						
NRAS	1.618	0.209～12.540	0.645	2.636	0.350～19.844	0.347
KMT2C	1.025	0.131～8.033	0.982	1.954	0.260～14.678	0.515
FLT3	3.975	0.851～18.564	0.079	1.639	0.376～7.137	0.510
PAX5	4.175	0.898～19.420	0.068	2.291	0.522～10.049	0.272
1个疗程诱导化疗疗效（非CR，CR）	2.979	1.450～6.118	0.003	1.981	1.017～3.861	0.045
移植与否（否，是）	6.377	2.097～19.392	0.001	3.421	1.313～8.914	0.012

注：CR：完全缓解

**表3:** 伴TP53基因突变急性B淋巴细胞白血病患者预后影响因素的多因素分析

因素	*HR*	95％ *CI*	*P*值
总生存			
FLT3突变	4.602	0.804～26.327	0.086
PAX5突变	2.695	0.368～19.725	0.329
1个疗程诱导化疗疗效（非CR，CR）	6.683	1.186～37.651	0.031
移植与否（否，是）	3.376	0.823～13.842	0.091
无事件生存			
染色体核型（复杂异常，其他）	1.860	0.681～5.080	0.226
1个疗程诱导化疗疗效（非CR，CR）	1.935	0.397～9.439	0.414
移植与否（否，是）	2.351	0.758～7.293	0.139

注：CR：完全缓解

## 讨论

人类TP53基因定位于17号染色体短臂，是一种重要的肿瘤抑制因子，该基因编码p53蛋白，在DNA修复、细胞周期阻滞、凋亡、衰老和自噬中发挥重要作用[4]–[5]。TP53突变在急性髓系白血病（AML）、ALL、骨髓增生异常综合征（MDS）及慢性淋巴细胞白血病（CLL）的检出率为10％左右[6]，在前体B-ALL中的检出率约为4.1％[7]。其中86％的突变集中在125和300密码子之间，主要对应于由外显子编码的DNA结合结构域，以错义突变最常见[8]。本研究中，共检测到36个TP53基因突变，其中2个位于DNA结合结构域4号外显子，34个位于DNA结合结构域第5～8号外显子，62％的患者为错义突变，突变位点与文献报道基本一致[9]–[10]。

既往研究表明TP53突变在复发或亚二倍体核型的ALL患者中发生率较高。Mühlbacker等[11]报道29例亚二倍体B-ALL患者，27例检出TP53突变。亚二倍体ALL预后差可能与TP53基因在该亚群中突变频率高有关[12]。也有研究表明伴有TP53突变的B-ALL中Ph染色体阳性率低[13]。本组479例B-ALL患者，伴有TP53基因突变的34例患者中仅2例伴有Ph染色体，Ph-like阳性1例；而445例不伴TP53突变患者172例伴有Ph染色体，Ph-like阳性13例；两组差异有统计学意义（*P*<0.001），与其一致。Salmoiraghi等[2]的研究表明，TP53基因突变并不影响成人ALL诱导缓解率，但与患者早期复发相关。本组34例伴TP53突变B-ALL患者接受IVP方案诱导化疗后仅1例未缓解，2例部分缓解，12例CR后巩固化疗过程中11例复发。表明伴TP53突变的B-ALL易早期复发。Forero-Castro等[7]研究发现伴TP53突变B-ALL患者5年OS率显著低于不伴TP53突变患者。这可能与伴TP53基因突变B-ALL患者诱导缓解后复发率高有关。

AML合并TP53基因突变应用地西他滨治疗有助于改善生存[14]。目前尚无地西他滨在TP53突变阳性B-ALL中应用的文献报道，这可能与CAR-T细胞输注在复发B-ALL中的疗效较好有关。2013年Brentjens等[15]首次报道了应用抗CD19 CAR-T细胞治疗5例复发成人B-ALL患者，均获得了缓解，MRD转阴。Pan等[16]总结了51例复发/难治B-ALL患者应用低剂量CAR-T细胞治疗的疗效，认为低剂量的CAR-T细胞输注治疗复发/难治B-ALL是安全有效的，进一步桥接allo-HSCT降低复发率。Zhang等[17]总结了110例高危B-ALL患者输注抗CD19 CAR-T细胞的疗效，伴TP53突变B-ALL在抗CD19 CAR-T细胞治疗缓解后仍有较高复发率。我们中心11例伴TP53基因突变患者在巩固化疗过程中复发，其中6例行抗CD19 CAR-T细胞治疗，4例缓解且MRD转阴，并在CAR-T细胞治疗缓解后桥接allo-HSCT，其中2例仍持续CR中，这与国内其他中心的研究结果基本一致。随后Pan等[18]对56例复发/难治儿童ALL患者行CAR-T细胞治疗，进一步研究CAR-T细胞治疗后复发的可能机制，研究发现伴TP53基因突变患者CAR-T细胞治疗后复发主要与靶抗原缺失有关。因此，对于伴TP53突变的高危B-ALL患者，复发后CAR-T细胞治疗清除MRD后择期行allo-HSCT应作为合理的选择。

高危B-ALL在allo-HSCT后仍有较高的复发率，输注供者来源的CAR-T细胞能获得较好的持续缓解。国内陈惠仁等[19]对7例allo-HSCT后复发患者输注抗CD19 CAR-T细胞，患者均获得MRD阴性的深层次缓解，其中5例单倍型移植患者采用供者来源。本研究16例伴TP53基因突变患者在CR_1_状态行allo-HSCT，3例移植后血液学复发，其中2例行供者来源的抗CD19 CAR-T细胞输注获CR_2_。对于allo-HSCT后复发的患者，选择供者来源的CAR-T细胞输注能再次获得缓解，但仍然面临CAR-T细胞治疗后再次复发的风险。这可能与CAR-T细胞在患者体内持续时间较短，白血病细胞靶抗原丢失有关，需要我们进一步探索研究。
